# Effects of Different Technological Forms of the *Perilla frutescens* in the Diet on Ruminal Fermentation, Milk and Plasma Fatty Acid Composition, Ruminal Biohydrogenation and Milk Quality in Dairy Goats

**DOI:** 10.1002/vms3.70087

**Published:** 2024-10-22

**Authors:** Selma Büyükkılıç Beyzi, İsmail Ülger, Emrah Kaya, Buğra Ocak, Yusuf Konca

**Affiliations:** ^1^ Department of Animal Science Faculty of Agriculture University of Erciyes Kayseri Turkey; ^2^ Department of Animal Science Faculty of Agriculture University of Iğdır Iğdır Turkey; ^3^ Department of Leather Engineering Faculty of Engineering Ege University Izmir Turkey

**Keywords:** biohydrogenation, cholesterol, conjugated linoleic acid (CLA), goat, omega 3, perilla

## Abstract

**Background:**

Fatty acids can be protected by changing their structure or form against microbial activity, and the different forms of fatty acids can modulate the ruminal biohydrogenation rate and contribute to the desired fatty acid profile in milk fat.

**Objectives:**

The study investigated the effects of perilla (*Perilla frutescens*) dietary supplementation in the diet in different technological forms (seed, oil and formaldehyde‐treated oil) on milk, plasma and ruminal fatty acid composition, and milk quality in lactating goats.

**Methods:**

The four dietary treatments consisted of (1) no supplementation, basal diet (CON); (2) perilla supplementation as seed at 44.7 g/kg (consisting of 20 g/kg oil (PS)); (3) perilla supplementation as oil at 20 g/kg (PO); (4) perilla supplementation as formaldehyde treated oil at 20 g/kg (protected perilla oil [PPO]). The experiment was implemented in a double 4 × 4 Latin square trial design, and sampling was carried out for 7 days after 21 days of adaptation.

**Results:**

Performance parameters were not affected by *P. frutescens* supplementation to the diet. PO decreased milk fat, whereas PPO increased milk fat. Milk cholesterol was not affected by *P. frutescens* dietary supplementation. Perilla oil supplementation in different forms to the diet did not affect ruminal pH, VFA and methane production. Perilla oil supplementation in different forms to the diet did not also affect the concentration of blood serum glucose, cholesterol and non‐esterified fatty acids. Perilla supplementation to the diet increased the milk conjugated linoleic acid (CLA), C18:3n‐3, C22:5n‐3, C20:5n‐3, C22:6n‐3 and polyunsaturated fatty acid (PUFA) concentrations, and PPO group showed the greatest values. Ruminal palmitic (C16:0) acid was decreased, and in perilla groups, stearic acid (C18:0) concentration had the lowest, and ruminal *c*‐9, *t*‐11 CLA concentration had the highest value in PPO.

**Conclusions:**

It has been found that the most effective form of perilla oil in increasing milk quality is that with formaldehyde treatment (protected form). Perilla oil, which is a rich source of omega 3 in the diet, can be used to increase milk quality in goats without adversely affecting performance, ruminal fermentation and blood parameters.

## Graphical abstract

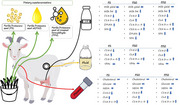

## 1 Introduction

In recent years, nutritionists have stated that some precautions should be taken because of the increase in the prevalence of nutritional‐based diseases. Scientists recommend the reduction of the amount of saturated fatty acids (SFA) and the increase in the amount of unsaturated fatty acids (UFA) in the diet (FAO 2010). Milk and dairy products contain high levels of SFA as a result of the saturation of fatty acids in the rumen through lipolysis and biohydrogenation processes. Therefore, although the fatty acid profile in the diet consists mostly of UFA, the fatty acid profile of lipids from the rumen consists mostly of SFA. For this reason, the rate of SFA is high in products obtained from ruminants.

Milk fat consists of approximately 70% SFA, 25% monounsaturated fatty acids (MUFA) and 5% polyunsaturated fatty acids (PUFAs) (Shingfield, Chilliard, and Toivonen 2008). In several studies, milk consumption has been associated with cardiovascular health problems due to its high rate of SFA (Elwood, Pickering, and Givens 2010; Astrup et al. 2011). However, many bioactive fatty acids in milk fat have been reported to have potential benefits for maintaining health status and preventing chronic diseases (Bauman and Lock 2010).

Omega‐3 fatty acids (n‐3) are essential for growth and development. They have many beneficial effects on human health and the prevention of chronic diseases such as cardiovascular, inflammatory and neurological (Yashodhara, Umakanth, and Pappachan 2009). It has been reported that 250–500 mg of total n‐3 fatty acids should be consumed daily in order to obtain these beneficial effects (Harris 2009). Therefore, it is important to increase the concentration of n‐3 fatty acids in milk fat.

Milk fat contains important intermediates such as conjugated linoleic acid (CLA). CLA is one of the intermediates of biohydrogenation; it is a positional and geometric isomer of linoleic acid. The *cis‐9, trans‐11* isomer of linoleic acid, is mostly known for its anticarcinogenic properties (Devery, Miller, and Stanton 2001; Durgam and Fernandes 1997). CLA is not produced by the human body. CLA prevents fat synthesis by blocking the lipoprotein lipase (LPL) enzyme and helps to reduce the amount of currently stored fat. For this reason, it is an important nutritional supplement recommended in the diet programme. However, CLA is known for its anti‐catabolic (prevention of muscle breakdown), antioxidant (reducing the effects of ageing and free radical damage), immune‐boosting, cholesterol‐lowering and anti‐cancer properties (Pariza 2004; Belury 2002). CLA especially reduces the risk of breast and prostate cancer, and atherosclerosis. In addition, CLA increases the sensitivity to insulin, increases the transition of fatty acids and glucose from adipose tissue to muscle tissue and provides a decrease in fat ratio (Ip, Singh, and Thompson 1994; Pariza, Park, and Cook 1999; Belury, Mahon, and Banni 2003). Although it is necessary to consume more than 400 mg of *cis‐9, trans*‐11 CLA per day to obtain these effects, the average daily amount of *cis‐9, trans*‐11 CLA in the body with general dietary habits is below 200 mg (Ritzenthaler, McGuire, and Falen 2001). For this reason, increasing the number of foods containing CLA is an important issue. When milk is evaluated in terms of CLA content, goat milk has the highest rate.

Changes in the diet are known as the best way to improve the fatty acid profile in products obtained from ruminants. However, there are many factors (dietary ingredients, fat source) that may affect the results (Büyükkılıç Beyzi, Gorgulu, and Kutlu 2020). Thus, it seems more effective to change only the fat sources while keeping all the other variables constant. Therefore, fat sources with different fatty acid profiles can be used in the diet. The various fat sources used to change the fatty acid composition of the diet, and the fatty acids in these sources can be protected against the biohydrogenation activity of the ruminal microbial population. It has been reported in studies that fatty acids can be protected by changing their structure or form against microbial activity (Jenkins and Bridges 2007). In previous studies, it has been reported that the protected forms of fatty acids provide increases in reaching the desired fatty acid profile in milk fat when compared to unprotected fatty acids (Jenkins and Bridges 2007).

*Perilla frutescens* is an annual herb belonging to the mint family (Lamiaceae) and predominantly produced in Asian countries. Although perilla seed is a good source of protein and rich in α‐linolenic acid (54%–64%) and linoleic acid (14%), it has recently received more attention due to its medicinal benefits and phytochemical contents (Dhyani, Chopra, and Garg 2019). In previous studies, perilla seed supplementation to rabbit diets increased the concentration of PUFA in muscles (Peiretti, Gasco, and Brugiapaglia 2011). In another study, meat quality was improved, but growth performance and carcass characteristics were not affected in lambs fed a diet supplemented with perilla seeds (Deng et al. 2018, 2017). This study hypothesizes that perilla seed may be a promising resource for improving product quality (fatty acid composition of its product) in animal nutrition. Moreover, little is known about the effect of adding perilla seed to ruminant diets on fatty acid composition. To the best of our knowledge, this is the first study focussing on the effects of perilla seed supplementation to the diet on the fatty acid composition of goat milk. The present study attempted to examine the effect of adding perilla in different forms (seed, oil and formaldehyde‐treated oil) on ruminal fermentation, milk, plasma, ruminal fatty acid composition and milk quality in lactating goats.

## 2 Materials and Methods

### 2.1 Animals, Diets and Study Design

Eight Aleppo goats were selected from a farm which has approximately 150 heads of dairy goats by considering age, breed, lactation number, number of offspring, live weight and milk yield. The four goats were cannulated to determine the ruminal fermentation and biohydrogenation; a total of eight dairy goats were used to determine the performance, milk quality and blood parameters. After 3 weeks of adaptation, their average milk yield was 3.2 ± 0.11 kg/day, and their live weight was 56 ± 0.27 kg. The diet was formulated to fulfil the National Research Council (2007) recommendations for the animals, and the dietary ingredients and nutritional composition are provided in Table 1. The diets were isocaloric and isonitrogenous. The four dietary treatments consisted of (1) no supplementation, basal diet (CON); (2) perilla supplementation as seed at 44.7 g/kg (consisting of 20 g/kg oil (PS); (3) perilla supplementation as oil at 20 g/kg (PO); (4) perilla supplementation as formaldehyde‐treated oil at 20 g/kg (protected perilla oil [PPO]).

**TABLE 1:** Dietary ingredients and chemical composition of the diet used in the experiment.

Ingredients (g/kg DM)	CON	PS	PO	PPO
Alfalfa hay	400.0	400.0	400.0	400.0
Barley	161.2	90.2	233.3	233.3
SSM 28	177.4	182.5	183.9	183.9
Wheat bran	18.8	27.5	27.6	27.6
Maize	241.9	254.4	134.5	134.5
Perilla seed	—	44.7	—	—
Perilla oil	—	—	20.0	—
Perilla oil (formaldehyde treated)	—	—	—	20.0
Salt	0.4	0.4	0.4	0.4
VMP	0.3	0.3	0.3	0.3
Chemical composition				
Dry matter (%)	89.22	89.09	89.14	89.03
Crude protein (%)	16.98	17.09	17.10	17.12
ADF (%)	18.10	17.54	15.35	15.38
NDF (%)	26.48	26.01	25.41	25.38
Ether extract (%)	2.42	4.45	4.44	4.46
NEL (Mcal/kg)	2.51	2.57	2.55	2.56
Fatty acid composition (g/100g)				
C4:0	1.30	0.39	0.40	0.40
C10:0	1.01	0.11	0.11	0.11
C14:0	2.01	0.10	0.10	0.09
C16:0	17.31	8.86	8.54	8.34
C16:1	2.46	0.14	0.14	0.15
C18:0	5.28	2.20	2.20	2.28
C18:1	26.33	19.80	19.61	19.39
C18:2	38.02	24.44	24.36	24.95
C18:3	3.77	43.96	43.03	43.90

Abbreviations: CON, control, diet without oil; PO, diet with perilla oil; PPO, diet with perilla rumen‐protected oil; PS, diet with perilla seed; SSM, sunflower seed meal; NEL, net energy for lactation; VMP, vitamin/mineral premix (Kavimix VM602 Kayseri, Turkey; 1 kg premix includes Vit‐D3, 1.500.000 IU; Vit‐A, 12.000.000 IU; Vit‐E, 30.000 mg; Se, 200 mg; Mn, 50.000 mg; Co, 200 mg; I, 800 mg; Fe, 50.000 mg; Zn, 50.000 mg; Cu, 10.000 mg).

The animals were housed in a closed‐type ovine unit in individual compartments (1 × 2 m^2^). The animals were fed twice a day, in the morning and evening. Feeds were prepared weekly. After 21 days of the experiment, the morning and evening milkings were separately weighed and taken for three consecutive milkings (23rd, 24th and 25th days), and the daily milk yield was determined. Rumen fluid was collected on the 3rd, 6th, 12th and 24th hours, including the 0th hour before feeding on the 26th day from cannulated goats. Some of the collected rumen fluid was stored at −20°C until analysis to identify the fermentation parameters. After the rumen fluids and milk samples obtained in the experiment were powdered using a freeze dryer, fatty acid analysis was performed. The cholesterol and fat content analyses were performed in fresh milk. On the 27th day of the experiment, blood was collected from each animal, and serum and plasma were obtained and kept at −80°C. Fresh milk samples were divided into two parts; one was frozen for fatty acid composition, and the other part was analysed for determination of milk fat.

### 2.2 Nutrient Analysis of Seeds, Feed Raw Materials and Diets

Dry matter (DM), crude protein (CP) and ether extract (EE) analyses were performed according to the methods specified in AOAC (1995).

### 2.3 Fatty Acid Composition Analysis in Milk, Feed and Rumen Fluid

All the samples were dried using a freeze dryer, and then oil was extracted. To this end, 0.375 g of dried sample was mixed with 15 mL of chloroform/methanol (2:1 vol/vol) and 375 µL of distilled water (Folch, Lees, and Sloane‐Stanley 1957). Afterwards, this extract was filtered, and 2.2 mL of distilled water was added, centrifuged at 800 × *g*, and a sample was collected from the upper phase. This sample was washed again (30 mL of chloroform, 480 mL of methanol and 470 mL of NaCl solution (7.3 g/L water)). After washing, approximately 3 mL of the oil‐containing phase was centrifuged again in order to remove other solvents, and the oil was purified.

From the extracted oil samples, 0.1–0.3 g was weighed, and 0.5 mL of 2 N methanolic KOH solution was added. This solution was made up to 10 mL with hexane. Then, this mixture was centrifuged at 4000 rpm for 10 min in a centrifuge instrument. At the end of the period, the tube was taken from the centrifuge instrument, and 1 mL of the upper phase of the solution was transferred into the vials (Fritsche and Steinhart 1998).

All the oil extracted and esterified samples taken into vials were placed in the autosampler of the gas chromatography (GC) instrument, and 1 µL of solution from each vial was injected into the GC, and the fatty acid profile was determined. FAMEs and CLA (AOAC 996.06 Standard, FAME Mix Cat No. 35077 and CLA Standard Cat No. 16413) standards were used. Fatty acids were analysed on a Shimadzu GC 2010 Plus instrument using a flame ionization detector (FID) and a fused silica column (J&W HP‐88 GC column, 100 m, 0.25 mm, 0.2 µm). Injection: 2.0 µL split (split ratio 200:1), 4 mm inlet liner (Cat No. 20814), injection temperature: 225°C, carrier gas: hydrogen, flow rate: 1.2 mL/min. The furnace temperature was adjusted from 100°C (4 min) to 240°C (10 min) at a rate of 3°C/min. As a result of GC analysis, the composition of fatty acids and their concentrations were determined from the curve obtained after reading the standards at certain concentrations.

### 2.4 Protection of Oils

Although there are many methods for protecting oils, the most preferred method in animal nutrition is to treat them with formaldehyde. According to the method reported by Petit (2003), seed oil was protected by adding 5.5 g of formalin (containing 37% formaldehyde) per kg of seed.

### 2.5 Determination of Fermentation Parameters in Rumen Liquor

The pH was immediately determined in the rumen fluids obtained in the in vivo stage, and then they were placed in ice water to prevent further digestion. The method of Erwin, Marco, and Emery (1961) was used to measure the concentration of volatile fatty acids in rumen fluid samples. After adding 1 mL of 25% metaphosphoric acid to the filtered samples, mixing well and waiting for 10 min, they were centrifuged for 10 min at 5000 rpm. After that, the syringe tip was passed through the filter (0.45 µm), placed in vials and kept in a deep freezer (−18°C) until it was injected into the GC instrument. In GC, acetic, propionic, butyric, valeric, isobutyric and isovaleric acid standards (WSFA‐2, Sigma‐Aldrich, Cat No. 47056) were prepared at different concentrations, and a standard curve was formed. Then, they were named on the basis of their retention times during the analysis and compared with the value calculated from the standard curve, and the amounts in the rumen fluid were calculated. The formula provided below was used to determine the methane production in the rumen.

Methane production (mmol/L) = 0.45 (acetate) − 0.275 (propionate) + 0.4 (butyrate); Moss, Jouany, and Newbold (2000).

### 2.6 Determination of Live Weight, Feed Intake and Milk Yield

To determine the live weights of the animals, they were individually weighed and recorded before the morning feeding by using a scale with ±100 g sensitivity with a cage on it. In the experiment, 20 kg of feed sacks for each animal was prepared to determine their feed intake. The sacks were marked with a group number, and the feeding of each animal was carried out according to this group number. During the experiment, daily feed intake was calculated with weekly measurements. In terms of the sensitivity of feed intake, the margin of error was minimized by including back weighing the feeds left in the feeders and spilt on the ground.

To determine the milk yield of the animals, automatic milking was performed by using the appropriate udder heads for the small cattle and connecting them to the milking hoppers. Milking was carried out twice, in the morning (08:00) and in the evening (20:00). The milk from each animal was weighed on a scale with ±2 g sensitivity, recorded separately in the morning and evening and collected to obtain daily milk yields.

### 2.7 Determination of the Fat and Cholesterol Ratio in Milk

Fat in milk was extracted from a 5 g milk sample using 100 mL of chloroform/methanol mixture (2:1, v/v) according to Folch, Lees, and Sloane‐Stanley (1957). The samples taken from the upper phase of the extract were obtained as residue by removing the solvent with the help of the evaporator. This process was repeated three times to collect all the oil. The oil percentage was calculated by weighing the obtained residue and proportioning it to the initial weight (5 g). A milk analyser (Milkana Multi‐Test, Air Milk Analyser) was used to determine milk composition.

Milk samples were analysed in GC using the cholesterol standard (Sigma‐Aldrich, Cat No. C8667) to determine the cholesterol ratio in milk. A standard was prepared at different concentrations, and a curve was calculated. The cholesterol ratio in milk was calculated by comparing it with this standard curve. An amount of 0.2 g of milk sample was weighed into a sample preparation tube to which 5 mL of methanolic KOH solution was added. The tube was tightly closed, and the contents were vortexed for 15 s. The lower half of the tube was then immersed in an 80°C bath and held there for 15 min, and the tubes were removed every 5 min and vortexed for 10 s. After heating, the tubes were cooled with tap water. After cooling, 1 mL of water and 5 mL of hexane were added, and the contents were vortexed for 1 min. It was then centrifuged at 2000 × *g* for 1 min. In GC, a silica column (60 m × 0.25 mm id) was used. The furnace temperature was set to 285°C, the injection port temperature was set to 300°C, and the FID temperature was set to 300°C. Flow rates were set at 2 mL/min for helium, 30 mL/min for hydrogen and 300 mL/min for air. The injection volume was 1 mL, a divided ratio of 20:1.

### 2.8 Determination of Glucose, Non‐Esterified Fatty Acid (NEFA) and Cholesterol in the Blood

For blood analysis, 10 mL of blood samples was collected from the vena jugularis vein in two different sterile tubes with and without anticoagulant during the last day of each treatment period and 4 h after feeding. Blood samples were centrifuged at 4000 × *g* for 20 min. The serum and plasma were collected and stored in cryo tubes at −80°C until analysis. Glucose was measured in a complete blood count instrument, and cholesterol and NEFA were performed in a spectrophotometer instrument (Gesan Chem 200) in accordance with the manufacturer's instructions with the help of a commercial tests kit (Gesan biochemical kits).

### 2.9 Determination of Fatty Acids in the Blood

The fat in the blood was extracted using *n*‐octane. The TAG fraction was separated using a silica column (Bond Elut SI, 500 mg, 3 mL; Varian Inc., Walnut Creek, CA, USA) and mixed with a mixture of hexane and methyl‐butyl‐ether (96:4 vol/vol). The TAG fraction in the solution was evaporated, and the fatty acids were separated into methyl esters with 0.4 mL of 0.5 N methanolic NaOCH_3_ (10 min at 80°C) and then incubated with 0.5 mL of 14% boron trifluoride (2 min at 80°C). It was converted to fatty acid esters with 100 µL of hexane and determined on a GC instrument.

### 2.10 Statistical Analysis

All data were subjected to ANOVA for a 4 × 4 Latin square design using the MIXED Models procedure using SPSS (IBM SPSS Statistics 22). The model included the fixed effects of period and treatment, the random effect of goat and the residual error. Data were reported as means ± SEM with *p* < 0.05 considered significant. Tukey's multiple comparison tests were used to determine the differences between means.

## 3 Results and Discussion

In the present study, the effects of using *P. frutescens* in different forms (seed, oil and preserved oil) in the diet on performance, milk and fatty acid composition, ruminal biohydrogenation and blood parameters in lactating goats were investigated. To the best of our knowledge, no studies have been found in the literature on the effect of perilla on milk quality. Hempseed and flaxseed are similar to (high n‐3 source) perilla in terms of fatty acid composition. For this reason, discussion was made by referring to mostly these oils and other unsaturated oil sources in the discussion section.

### 3.1 Performance

The live weight, feed intake and milk yield were not affected by the treatments (*p* > 0.05) (Table 2). The use of perilla oil in different forms in the diet did not affect performance parameters such as feed intake and milk yield in lactating goats. Similarly, it was reported that feed intake was not affected by the addition of 2% and 4% soy and flaxseed oil to the diet (Bu, Wang, and Dhiman 2007; Ye et al. 2009). In a study, it was reported that the use of 2% linseed oil and its protected form (encapsulated) in the diet reduced feed intake in goats (Kim, Lee, and Cho 2020). This has been explained by the fact that UFA inhibits microbial metabolism in the rumen and reduces NDF digestibility, resulting in lower DM intake in fat‐containing feeds (Jenkins 1993). Furthermore, Morsy, Kholif, and Kholif (2015) stated that daily feeding of 50 g of seeds or 20 mL doses of sunflower oil did not affect feed intake in lactating goats. In other studies, it has been reported that the addition of fat to the diet causes a decrease in milk production (Ahnadi, Beswick, and Delbecchi 2002; Boeckaert et al. 2008) or does not affect it (Allred, Dhiman, and Brennand 2006; Stamey et al. 2012). These detected differences may vary depending on some factors such as the amount of these sources used in the diet, the form of the oil, the length of the trial period or the composition of the basal diet.

**TABLE 2:** The effects of perilla seed, oil and rumen‐protected oil supplementation to the diet on live weight, feed intake and milk yield in goats.

Parameters	CON	PS	PO	PPO	SEM	*p*
Live weight (kg)	55.23	54.83	56.72	56.31	0.061	0.254
Feed intake (g/day)	1987.1	1971.5	1951.6	1901.4	64.05	0.758
Milk yield (g/day)	2441.2	2468.7	2341.2	2496.4	112.3	0.644

Abbreviations: CON, control, diet without oil; PO, diet with perilla oil; PPO, diet with perilla rumen‐protected oil; PS, diet with perilla seed; SEM, standard error mean.

### 3.2 Milk Composition

The use of perilla seeds, oil and protected fat in the diet did not affect the concentration of non‐fat DM, protein, lactose, pH and cholesterol in goat milk (*p* > 0.05; Table 3). However, the use of perilla as oil in diet decreased the fat content in milk, whereas its use in protected form increased the fat rate in milk (*p* < 0.05). In a study investigating the use of hemp as seeds or pulp in the diet, it was reported that its addition significantly increased milk and milk fat yield (Mierlita 2018). In a study, milk production increased but milk fat content decreased by approximately 12% with the use of flaxseed or its oil in the diet (Kholif, Morsy, and Abdo 2018). Similarly, Morsy, Kholif, and Kholif (2015) reported that the concentration of milk fat increased with the addition of 50 g of sunflower seeds per day or 20 mL of sunflower seed oil per day to the diet. It has been reported that the addition of sunflower oil to cow rations increases milk yield (Abu Ghazaleh and Holmes 2007; Castro, Manso, and Jimeno 2009). However, although other studies observed a decrease in milk production with sunflower seeds (Petit, Germiquet, and Lebel 2004; Mohammed, McGinn, and Beauchemin 2011), no change was reported with the addition of sunflower oil (Ollier, Leroux, and de la Foye 2009) or sunflower seeds (Beauchemin, McGinn, and Benchaar 2009). In another study, it was found that the use of flaxseed instead of flaxseed oil in the diet decreased the milk fat content, which might be attributed to the increased linoleic and linolenic fatty acids and ruminal biohydrogenation (Kholif, Morsy, and Abdo 2018). Similarly, lower milk fat content was found in lactating cows fed a diet supplemented with 20 g/kg linseed oil in DM (Ye et al. 2009). Dietary fat sources rich in long‐chain UFAs reduce milk fat content because of their adverse effects on cellulose digestion and ruminal acetate concentration (Onetti, Shaver, and McGuire 2001). Moreover, there may be another reason, and adding PUFA‐rich fat to the diet can decrease de novo lipogenesis (Chilliard, Ferlay, and Doreau 2001). The reasons for the inconsistency of the results may be the type of oil, the form used, the dosage and the fatty acid content.

**TABLE 3:** The effects of perilla seed, oil and rumen‐protected oil supplementation to the diet on the composition of goat milk.

Parameters (g/kg)	CON	PS	PO	PPO	SEM	*p*
Fat	5.18b	5.00b	4.44c	5.41a	0.05	0.042
Non‐fat dry matter	8.35	8.67	8.10	8.23	0.19	0.759
Protein	3.44	3.70	3.53	3.35	0.13	0.815
Lactose	4.28	4.30	4.26	4.26	0.02	0.803
pH	5.74	5.73	5.73	5.74	0.00	0.627
Cholesterol (mg/100 g)	25.81	25.80	25.78	25.88	0.02	0.464

Abbreviations: CON, control, diet without oil; PO, diet with perilla oil; PPO, diet with perilla rumen‐protected oil; PS, diet with perilla seed; SEM, standard error mean.

Values in the same row with superscript letters are significantly different at *p* < 0.05.

### 3.3 Milk Fatty Acid Composition

The effect of perilla seed, oil and rumen‐protected oil supplementation to the diet on fatty acids composition in goat milk is shown in Table 4. It was clear that C4:0, C6:0, C15:0, C16:0 and C18:0 fatty acids were affected by the treatments (*p* < 0.05), but the ratio of other UFA was not affected by the treatments (*p* > 0.05). The ratios of C4:0, C6:0 and C15:0 fatty acids were the highest in the group with the PPO, whereas C16:0 and C18:0 fatty acids decreased in the perilla groups (*p* < 0.05). Supplementing PUFA‐rich oil sources (e.g., flaxseed) to diets can inhibit the synthesis of de novo FAs (C4:0‐16:0) in the mammary gland by inhibiting or reducing enzymes in de novo lipogenesis pathways, including palmitic acid (Akraim, Nicot, and Juaneda 2007; Cívico et al. 2017). Decreased SFAs, especially C16:0, linked to increased low‐density lipoprotein (LDL) in plasma, can provide nutritional benefits from a human health perspective (Haug, Høstmark, and Harstad 2007; Mills, Ross, and Hill 2011). Stearic acid (C18:0) in milk is an 18‐chain SFA with no double bonds. It is derived from the transfer of double‐linked 18‐chain fatty acids to the mammary glands after biohydrogenation by microorganisms in the rumen or induction of stearic acid in feed (Kim, Lee, and Cho 2020). In the current study, the use of perilla oil in different forms did not affect the stearic acid concentration in milk among the treatment groups. In a study, a lower C18:0 ratio was determined in milk fat obtained from animals fed a diet containing formaldehyde‐treated flaxseed (Petit 2002).

**TABLE 4:** The effects of perilla seed, oil and rumen‐protected oil supplementation to the diet on milk fatty acids in goats.

Fatty acids (g/100 g)	CON	PS	PO	PPO	SEM	*p*
Saturated fatty acids						
C4:0	2.82^ab^	2.98^ab^	2.43^b^	3.15^a^	0.10	0.038
C7:0	0.14	0.22	0.26	0.29	0.02	0.084
C6:0	2.80^ab^	2.80^ab^	2.61^b^	2.96^a^	0.04	0.027
C10:0	5.99	5.36	5.23	6.01	0.30	0.118
C12:0	2.61	2.31	1.82	2.30	0.12	0.105
C13:0	0.05	0.05	0.06	0.07	0.00	0.204
C14:0	6.68	6.59	6.08	6.01	0.28	0.730
C15:0	0.78^ab^	0.74^b^	0.81^ab^	1.02^a^	0.04	0.034
C16:0	23.32^a^	19.16^b^	19.93^b^	19.34^b^	0.36	0.011
C17:0	0.53	0.28	0.44	0.42	0.05	0.327
C18:0	13.75^a^	11.89^b^	12.27^b^	11.32^b^	0.32	0.027
C20:0	0.20	0.15	0.13	0.12	0.01	0.198
C22:0	0.06	0.06	0.06	0.06	0.02	0.128
Monounsaturated fatty acids						
*c*‐9 C10:1	0.22	0.16	0.59	0.41	0.07	0.081
*t*‐9 C12:1	0.04	0.03	0.03	0.04	0.00	0.639
*c*‐9 C14:1	0.16^b^	0.21^a^	0.19^a^	0.25^a^	0.01	0.041
*c*‐9 C16:1	0.18	0.37	0.31	0.40	0.04	0.100
*t*‐10 C18:1	0.35	0.11	0.27	0.20	0.03	0.060
*t*‐12 C18:1	0.38	0.46	0.80	0.99	0.11	0.153
*c*‐12 C18:1	0.47^ab^	0.78^a^	0.18^b^	0.38^ab^	0.07	0.006
*t*‐13, 14 C18:1	0.06	0.10	0.04	0.52	0.11	0.403
*t*‐9 C18:1	0.08	0.09	0.11	0.09	0.01	0.515
*t*‐15, *c*‐10 C18:1	0.33	0.28	0.37	0.14	0.04	0.133
*t*‐11 C18:1	1.16^c^	3.84^b^	3.76^b^	5.47^a^	0.27	0.002
*c*‐9 C18:1	30.05^b^	31.96^a^	31.94^a^	26.91^c^	1.08	0.035
*c*‐11, 12 C18:1	0.16^c^	0.25^ab^	0.18^bc^	0.27^a^	0.01	0.005
*t*‐6, 8 C18:1	0.09^c^	0.21^bc^	0.34^ab^	0.52^a^	0.05	0.000
Polyunsaturated fatty acids						
C18:2n‐6	3.46^a^	2.48^b^	2.69^b^	2.29^b^	0.13	0.001
*t*‐9, *t*‐12 C18:2	0.05	0.09	0.10	0.09	0.01	0.287
*c*‐9, *t*‐12 C18:2	0.09	0.11	0.14	0.13	0.01	0.504
*t*‐9, *c*‐12 C18:2	0.74	0.85	0.89	0.82	0.06	0.886
*c*‐9, *t*‐11 CLA	1.02^b^	1.39^ab^	1.41^ab^	1.86^a^	0.10	0.013
*t*‐10, *c*‐12 CLA	0.06^ab^	0.26^ab^	0.05^b^	0.31^a^	0.04	0.016
*c*‐9, *c*‐12 CLA	0.03^b^	0.09^ab^	0.04^ab^	0.09^a^	0.01	0.030
Sum of fatty acids						
C18:3n‐3	0.24^b^	2.16^a^	2.45^a^	2.71^a^	0.06	0.016
C20:3n‐6	0.12	0.09	0.08	0.07	0.01	0.112
C22:2n‐6	0.20	0.16	0.15	0.21	0.01	0.117
C22:5n‐3	0.20^d^	0.36^b^	0.28^c^	0.41^a^	0.02	0.000
C20:4n‐6	0.19	0.15	0.15	0.15	0.01	0.135
C20:5n‐3	0.06^b^	0.20^a^	0.09^b^	0.25^a^	0.02	0.000
C22:6n‐3	0.08^b^	0.17^ab^	0.24^a^	0.25^a^	0.02	0.022
Σ*trans* C18:1	2.45^c^	5.09^b^	5.69^b^	7.63^a^	0.42	0.001
ΣC18:1	31.01^ab^	33.27^a^	32.67^a^	28.70^c^	1.06	0.039
ΣC18:2	5.45	5.27	5.52	5.59	0.13	0.908
ΣCLA	1.11^c^	1.74^b^	1.70^b^	2.26^a^	0.13	0.019
ΣSFA	59.73^a^	52.59^b^	51.93^c^	53.07^b^	0.19	0.047
ΣMUFA	33.73^b^	38.85^a^	39.11^a^	37.29^ab^	0.94	0.044
ΣPUFA	6.54^c^	8.56^b^	8.96^b^	9.64^a^	0.16	0.045
Σn‐3/n‐6	0.15^b^	1.00^b^	1.00^b^	1.33^a^	0.05	0.001
Δ‐9 desaturase index						
C14:1/C14:0	0.02^b^	0.03^a^	0.03^a^	0.04^a^	0.00	0.053
C16:1/C16:0	0.01	0.02	0.02	0.02	0.00	0.216
*c*‐9 C18:1/C18:0	2.19^c^	2.69^a^	2.60^a^	2.38^b^	0.10	0.013
*c*‐9, *t*‐11 C18:2/*t*‐11 C18:1	0.88^a^	0.36^b^	0.38^b^	0.34^b^	0.01	0.003

Abbreviations: CLA, conjugated linoleic acid; CON, control, diet without oil; MUFA, monounsaturated fatty acids; PO, diet with perilla oil; PPO, diet with perilla rumen‐protected oil; PS, diet with perilla seed; PUFA, polyunsaturated fatty acids; SEM, standard error mean; SFA, saturated fatty acids.

Values in the same row with superscript letters are significantly different at *p* < 0.05.

The *cis‐9* C10:1*, trans‐9* C12:1, *cis‐9* C16:1, *trans‐10* C18:1, *trans‐12* C18:1, *trans‐13, 14* C18:1, *trans‐9* C18:1 and *trans‐15, 10* C18:1 fatty acids were not affected by the treatments (*p* > 0.05). *Cis‐9* C14:1 fatty acid increased in perilla‐added groups (*p* < 0.05). *Cis‐12* C18:1 fatty acid was higher in the PS group (*p* < 0.05). The *cis‐9* C18:1 was highest in the PS and PO groups and lowest in the PPO group (*p* < 0.05). *Cis‐11, 12* C18:1 and *trans‐6, 8* C18:1 fatty acids were found to be the highest in the PPO group and the lowest in the control group (*p* < 0.01). *Trans‐11* C18:1 fatty acid was found to be higher in perilla groups compared to the control group, whereas the highest value was found in the PPO group (*p* < 0.01). The amount of UFA in the diet and their source and accessibility by microorganisms increase the extent of ruminal biohydrogenation and cause the formation of different intermediates in milk fat (Chilliard, Glasser, and Ferlay 2007). The findings of the present study indicated that the highest concentration of *trans*‐11 C18:1 fatty acid, which is one of the most important intermediates, increased in perilla groups. In a study conducted with sheep, the use of fish oil in the diet in protected (formaldehyde + casein) form increased the ratio of C18:1 *trans* isomers and fatty acid derivatives, which pointed to its interaction with ruminal metabolism (Kitessa, Gulati, and Ashes 2001).

The *trans‐9, trans‐12, cis‐9, trans‐12, trans‐9, 12* C18:2, C20:3n‐6, C22:2n‐6 and C20:4 were not affected by perilla dietary supplementation (*p* > 0.05). C18:2 fatty acid content was decreased in the perilla‐supplemented groups (*p* < 0.01). *Cis‐9, trans‐11, cis‐9, 12* and *trans‐10, 12* CLA fatty acids were found to be the highest in the PPO group (*p* < 0.05). Similarly, the C18:3, C22:5, C20:5 and C22:6n‐3 fatty acids concentrations were highest in the PPO group (*p* < 0.01). The ΣC18:2 and C16:1/C16:0 concentrations in milk fat obtained from goat's milk were not affected by the treatment (*p* > 0.05). It was determined that the total *trans* C18:1, total CLA, n‐3/n‐6 and total PUFA fatty acids were highest in the PPO group (*p* < 0.05). Total *cis‐*C18:1 fatty acid was found highest in the PS and PO groups and lowest in the PPO group (*p* < 0.05).

CLA in milk originates mainly from rumen biohydrogenation of linoleic acid (Harfoot and Hazlewood 1997) or from mammary gland synthesis (Corl, Baumgard, and Dwyer 2001). Therefore, an increased milk CLA concentration can be obtained with a diet enriched with linoleic and linolenic acid sources (Bu, Wang, and Dhiman 2007; Ye et al. 2009; Morsy, Kholif, and Kholif 2015; Kholif, Morsy, and Abd El Tawab 2016). The addition of 18:3n‐3 fatty acids to ruminant diets may lead to CLA production by inducing the isomerization of fatty acids by rumen microorganisms (Doreau and Ferlay 1994). In a study, cows fed flaxseed with fish oil had a lower *trans*‐11 C18:1 ratio compared to flaxseed fed as oil. However, with increasing *trans*‐11 C18:1 concentration, the concentration of *trans*‐10 C18:1 increases as rumen microorganisms change their biohydrogenation pathways accordingly (Shingfield et al. 2003; Shingfield, Reynolds, and Hervás 2006). Contrary to the *trans*‐11 C18:1 ratio in the present study, the *trans*‐10 C18:1 fatty acid concentration was not affected by changes in the diet. In a study, it was reported that the CLA content in milk did not change with the use of flaxseed or oil in the diet and even decreased numerically with the use of oil, whereas it increased with the microencapsulation of flaxseed oil compared to the control (Kim, Lee, and Cho 2020). In a study investigating the use of hemp as seed or pulp in the diet, it was found that the addition of hemp resulted in an increase in *trans*‐11 C18:1 (vaccenic acid), CLA and especially *c*‐9, *t*‐11 CLA isomer in milk fat. It was reported that it produced more α‐linolenic acid, vaccenic acid and total CLA in milk fat compared to the group (Mierlita 2018). In the present study, it was determined that the amount of α‐linolenic acid and n‐3 PUFA in milk fat increased with the use of perilla seed oil in different forms in the diet. Similarly, in a study, it was reported that the use of hemp in the diet as seeds or pulp increased the amount of n‐3 PUFA in milk fat (Mierlita 2018).

In terms of total fatty acids, the use of perilla oil in different forms in the diet decreased the SFA ratio in milk and increased the PUFA, n‐3/n‐6 and total CLA concentrations. This effect was more intense in the rumen‐protected oil group. These results are attributed to the increased amount of unsaturated fat released into the rumen and absorbed in the small intestine due to the use of fat sources with a high C18:3n‐3 fat ratio in the diet. The PUFA is not synthesized by ruminants, so their concentration in milk depends on the amount of PUFA absorbed from the intestines. In a study, it was stated that the use of omega‐3 sources in the diet could increase the amount of n‐3 that reaches the small intestine and is absorbed (Nafikov, Soyeurt, and Beitz 2015). Supplementation of fat sources rich in PUFA to the diet inhibits de novo synthesis of milk fatty acids and reduces total SFA content (Cívico et al. 2017) by competing for esterification with short‐chain fatty acids synthesized in the mammary gland. Moreover, the inhibitory effect of *trans*‐18 isomers produced during biohydrogenation on the de novo synthesis of SFA is another reason for the low SFA concentration (Chilliard, Ferlay, and Doreau 2001). Studies have shown that the addition of seeds or oil to the diet increased the PUFA and MUFA content of milk fat in goats, and there was a decrease in the SFA content (Castro, Manso, and Jimeno 2009; Mohammed, McGinn, and Beauchemin 2011).

The C14:1/C14:0 and *cis‐9* C18:1/C18:0 of Δ‐9 desaturase index were increased, whereas the *cis‐9, trans‐11* C18:2/*trans*‐11 C18:1 index decreased in milk fat by the dietary supply of perilla in the current experiment of dairy goats in the current study. The concentrations of *cis*‐9 14:1 and *cis*‐9 16:1 affect the endogenous synthesis of Δ‐9 desaturase and may cause an increase in biohydrogenation intermediates reaching the mammary gland. Bernard, Leroux, and Chilliard (2013) suggest that the activities of lipogenic enzymes were regulated by UFA in the diet. In a study, an increase in the level of *trans*‐9 16:1 in milk fat was reported with the addition of flaxseed to the diet (Luna, Rodríguez‐Pino, and De la Fuente 2009; Bernard et al. 2009). In a study, it was shown that *trans*‐11 C18:1 reflected the use of 9‐desaturase in the synthesis of *cis*‐9, *trans*‐11 CLA, thus causing a *trans*‐11 C18:1 fatty acid change in milk (Corl, Baumgard, and Dwyer 2001). In another study, it was shown that the addition of *cis*‐9 bonds to *trans*‐12 C18:1 produced *cis*‐9, *trans*‐12 C18:2 found in milk fat (Griinari, Corl, and Lacy 2000).

### 3.4 Ruminal Biohydrogenation

The effects of perilla seed, oil and rumen‐protected oil supplementation to the diet on ruminal fatty acids at 24th hours are presented in Table 5. The effect of the use of perilla oil forms in the diet on the concentration of ruminal *trans*‐10 C18:1, C22:5n‐6 and C22:6n‐3 fatty acids was not significant (*p* > 0.05). C14:0, *trans*‐6,7,8 C18:1, *trans*‐9 C18:1, *trans*‐12 C18:1, *cis*‐11, *trans*‐15 C18:1, *cis*‐13 C18:1, *cis*‐14, *trans*‐16 C18:1 and *trans*‐10, *cis*‐12 CLA fatty acids increased in perilla groups (*p* < 0.05). C16:0, *cis*‐12 C18:1 and C18:2n‐6 fatty acids decreased in perilla groups (*p* < 0.05). Although C18:0 fatty acid was highest in the control group, it was found to be the lowest in the PPO group (*p* < 0.05). *Trans*‐11 C18:1 fatty acid was highest in the PPO group and lowest in the control group (*p* < 0.01). *Trans*‐13 and *trans*‐14 C18:1 fatty acids were highest in the PS and PPO groups and lowest in the control group (*p* < 0.01). Total C18:1 was highest in the PO group and lowest in the PPO group (*p* < 0.01). C18:3n‐3 fatty acid ratio was highest in the PPO group and lowest in the control group (*p* < 0.01).

**TABLE 5:** The effects of perilla seed, oil and rumen‐protected oil added to the diet on rumen fluid fatty acids in lactating goats.

Parameters (g/100g)	CON	PS	PO	PPO	SEM	*p*
C14:0	1.75^b^	2.04^a^	2.31^a^	2.24^a^	0.18	0.000
C16:0	14.16^a^	13.44^b^	13.91^b^	13.84^b^	0.24	0.000
C18:0	42.15^a^	33.25^c^	38.05^b^	30.94^d^	1.41	0.000
*t*‐6,7,8 C18:1	0.06^b^	0.50^a^	0.61^a^	0.65^a^	0.03	0.000
*t*‐9 C18:1	0.19^b^	0.39^a^	0.35^a^	0.38^a^	0.04	0.000
*t*‐10 C18:1	0.45	0.50	0.59	0.60	0.06	0.248
*t*‐11 C18:1	1.45^d^	4.52^b^	3.62^c^	5.58^a^	0.11	0.000
*t*‐12 C18:1	0.31^b^	0.63^a^	0.61^a^	0.64^a^	0.04	0.000
*t*‐13, *t*‐14 C18:1	0.44^c^	1.71^a^	1.56^b^	1.74^a^	0.06	0.000
*c*‐11, *t*‐15 C18:1	1.45^b^	2.41^a^	2.06^a^	2.59^a^	0.08	0.000
*c*‐12 C18:1	0.39^a^	0.20^b^	0.19^b^	0.20^b^	0.02	0.000
*c*‐13 C18:1	0.08^b^	0.25^a^	0.27^a^	0.29^a^	0.02	0.000
*c*‐14, *t*‐16 C18:1	0.24^b^	0.42^a^	0.38^a^	0.45^a^	0.02	0.019
Total C18:1	6.20^c^	7.14^b^	8.41^a^	5.80^d^	0.25	0.000
*c*‐9, *t*‐11 CLA	0.14^d^	0.38^b^	0.31^c^	0.54^a^	0.01	0.000
*t*‐10, *c*‐12 CLA	0.03^b^	0.13^a^	0.15^a^	0.15^a^	0.03	0.000
C18:2n‐6	2.65^a^	1.91^b^	1.92^b^	1.89^b^	0.08	0.000
C18:3n‐3	0.58^c^	1.94^b^	2.03^b^	2.29^a^	0.02	0.000
C22:5n‐6	0.03	0.02	0.03	0.03	0.00	0.258
C22:6n‐3	0.04	0.04	0.04	0.04	0.00	0.441

Abbreviations: CLA, conjugated linoleic acid; CON, control, diet without oil; PO, diet with perilla oil; PPO, diet with perilla rumen‐protected oil; PS, diet with perilla seed; SEM, standard error mean.

Values in the same row with superscript letters are significantly different at *p* < 0.05.

With the supplementation of perilla oil in different forms in the diet, the C18:0 stearic acid decreased compared to the control in the ruminal fluid. There was a significant increase in the intermediate products formed as a result of biohydrogenation. The decrease in the concentration of C18:0 fatty acid, which is formed by the biohydrogenation of 18‐carbon UFAs in the diet, in milk of the treatment groups; it is observed that the biohydrogenation of the UFA is not completed with the increase in biohydrogenation intermediates. The concentration of fatty acids detected in the rumen fluid transferred to the milk was compatible with the fatty acid profile in milk. In the current study, it was determined that the best practice in terms of treatments was in the rumen‐protected oil group, followed by the seed group. Formaldehyde treatment of oilseeds before use in the diet has been shown in studies to reduce biohydrogenation of C18:3n3 fatty acid in the rumen (Sterk, Hovenier, and Vlaeminck 2010). In a study aimed at protecting PUFA (n‐3) from biohydrogenation in the rumen, flaxseed was pretreated with sodium hydroxide, formaldehyde, formic acid and ammonium tetraformate. It was concluded that pretreatment using formic acid followed by treatment with formaldehyde provided the best protection against ruminal microbial biohydrogenation (Sinclair et al. 2005). Similarly, it has been reported that technological processes such as technological pretreatment, formaldehyde and extrusion applied to oilseeds increase the rates of biohydrogenation intermediates in the rumen (Bayourthe, Enjabert, and Moncoulon 2000; Chouinard, Corneau, and Butler 2001; Enjalbert, Eynard, and Nicot 2003; Akraim, Nicot, and Juaneda 2007). Gonthier, Mustafa, and Ouellet (2005) reported that chemical treatments such as formaldehyde treatment were more effective than heat treatment in protecting the UFA from ruminal biohydrogenation. In their in vitro study, Sinclair et al. (2005) claimed that pretreatment of flaxseed with sodium hydroxide or formic acid followed by treatment with formaldehyde resulted in effective protection of C18:3n3 in the rumen. In a study, it has been reported that cows fed with ground flaxseed, formaldehyde‐treated linseed oil, extruded flaxseed and DHA‐combined flaxseed had low C18:3n‐3. It is also reported that the degree of biohydrogenation is lower in cows fed with extruded flaxseed (Sterk et al. 2011).

Moreover, the findings of the present study demonstrated that *t*‐11 C18:1 (vaccenic acid) fatty acid, which is an important CLA isomer, was increased in the treatment groups, and there was a significant increase with formaldehyde application. Vaccenic acid is known as the CLA isomer, and several studies have shown that it is associated with preventing cancer, strengthening the immune system and reducing cardiovascular diseases (Whigham, Cook, and Atkinson 2000; Belury 2002; Pariza 2004). In the current study, the total CLA concentration is largely due to *c‐9, t‐11* (rumenic acid) and *t‐10*, *c‐12* fatty acid isomers in total CLA. It was determined that these fatty acids increased in the treatment groups. The production of biohydrogenation intermediates is important because it plays a key role in mammary gland lipogenesis (Bauman and Griinari 2003) and especially in the de novo synthesis of fatty acids (Chilliard and Ferlay 2004). The findings further indicate that the concentration of vaccenic acid (*t‐11* C18:1) increases with the addition of 2% and 3% flaxseed to the diet (Ding et al. 2016). C18:3 fatty acid increased in the treatment groups, and a significant increase was achieved in the perilla oil group, which was applied with formaldehyde. Caroprese, Marzano, and Marino (2010) observed that the whole flaxseed supplementation to the diet increased the C18:3n‐3 fatty acid in milk. Petit (2003) reported higher levels of C18:3n‐3 in milk in cows fed whole flaxseed compared to cows fed control, whole sunflower seeds and its Ca salts.

### 3.5 Ruminal Fermentation

The effect of perilla oil supplementation in different forms to the diet on the 24th hour ruminal fermentation parameters is presented in Table 6. The effects of the supplementation of perilla oil forms in the diet on ruminal pH, acetate, propionate, iso‐butyrate, butyrate, iso‐valerate, valerate and acetate/propionate parameters, and methane production were not significant (*p* > 0.05).

**TABLE 6:** The effects of perilla seed, oil and rumen‐protected oil supplementation to the diet on ruminal fermentation parameters in lactating goats.

Parameters (mmol/100 mmol)	CON	PS	PO	PPO	SEM	*p*
pH	6.05	6.12	6.10	6.05	0.12	0.457
Acetate	66.45	66.28	66.29	66.34	0.09	0.441
Propionate	17.35	17.42	17.51	17.41	0.07	0.256
*iso‐*Butyrate	1.12	1.19	1.06	1.09	0.01	0.399
Butyrate	11.47	11.49	11.52	11.54	0.01	0.896
*iso‐*Valerate	1.80	1.85	1.83	1.83	0.01	0.101
Valerate	1.81	1.77	1.79	1.79	0.01	0.196
Acetate/Propionate	3.83	3.80	3.79	3.81	0.01	0.352
Methane production	28.81	28.45	28.54	28.61	0.03	0.456

Abbreviations: CON, control, diet without oil; PO, diet with perilla oil; PPO, diet with perilla rumen‐protected oil; PS, diet with perilla seed; SEM, standard error mean.

In the current study, although the rumen pH changed to 6.05–6.12, it was not affected by the treatments. Acceptable values of pH for the digestion of cellulose in the rumen range from 5.85 to 6.14 (Ryle and Orskov 1990). Oils added to feed can increase rumen acidity and cause lower ruminal pH (Staples 2006). In a study, the addition of flaxseed oil reduced ruminal pH by 4.6% (Kholif, Morsy, and Abdo 2018). In similar studies, it was reported that ruminal pH values decrease with the addition of oil to goat diets (Morsy, Kholif, and Kholif 2015; Kholif, Morsy, and Abd El Tawab 2016). In a study, it was reported that the addition of 30 g/kg flaxseed oil to the diet did not change the ruminal pH (Zhang, Mustafa, and Zhao 2006). The use of different forms of perilla oil in the diet did not affect the ruminal acetate, propionate, iso‐butyrate, butyrate, iso‐valerate, valerate and acetate/propionate ratios. Production of volatile fatty acids in the rumen is dependent on many factors, including nutrient, rate of absorption, rate of digestion from the rumen and activity of the microbial population in the rumen (Flatt, Warner, and Loosli 1956). In a study comparing the use of flaxseed oil and flaxseed in the diets of goats, flaxseed oil decreased the rumen pH and acetate ratio, whereas flaxseed and flaxseed oil increased the total volatile fatty acid content and propionate concentration. Although flaxseed oil had no effect on cellulose digestibility, flaxseed increased the digestibility of cellulose (Kholif, Morsy, and Abdo 2018). In a study, it was reported that the fish oil (increasing levels) supplemented diet increased the pH and propionate in the rumen and decreased the acetic acid concentration (Boeckaert et al. 2008). Morsy, Kholif, and Kholif (2015) stated that ruminal acetate content increased with feeding with seeds and oil. The addition of flaxseed (alone or in combination with soybean oil) resulted in an increase in the total volatile fatty acid concentration and an increase in propionate with flaxseed supplementation compared to the control, where there was no difference in total volatile fatty acid concentration with the dietary use of flaxseed and soybean oil (Kholif, Morsy, and Abd El Tawab 2016). In a study, it was reported that the addition of 30 g/kg flaxseed oil to the diet did not affect the ruminal pH, individual and total VFAs (Zhang, Mustafa, and Zhao 2006). According to Côrtes et al. (2010), the use of 4.1% flaxseed in the diet had no effect on the total VFA concentration and the molar proportions of individual VFAs. However, Gonthier, Mustafa, and Berthiaume (2004) reported that the use of higher levels of flaxseed (12.5% of the diet) decreased the acetate ratio and increased the propionate ratio. Consistent with this finding, Soder, Brito, and Rubano (2013) reported a decrease in the acetate/propionate ratio when 10% flaxseed was added to a forage‐based diet. For this reason, it is recommended that the use of oilseeds in the diet should not exceed 10%. It was determined that methane production was not affected using perilla oil in different forms in the diet. In a study, it was reported that crushed flaxseed and flaxseed oil in the diet caused an increase in propionate by causing the inhibition of methanogens in the rumen (Patra and Yu 2012).

### 3.6 Blood Serum Parameters

The effects of perilla seed, oil and rumen‐protected oil supplementation to the diet on blood serum parameters are shown in Table 7. The supplementation of perilla oil forms in the diet did not affect the concentration of blood serum glucose, cholesterol and NEFA (*p* > 0.05).

**TABLE 7:** The effects of perilla seed, oil and rumen‐protected oil supplementation to the diet on blood serum parameters in lactating goats.

Parameters, mg/dL	CON	PS	PO	PPO	SEM	*p*
Glucose	76.38	69.43	77.62	69.88	1.46	0.076
Cholesterol	76.28	75.40	76.95	72.01	1.61	0.751
NEFA	1.01	1.03	1.04	1.03	0.03	0.254

Abbreviations: CON, control, diet without oil; NEFA, non‐esterified fatty acids; PO, diet with perilla oil; PPO, diet with perilla rumen‐protected oil; PS, diet with perilla seed; SEM, standard error mean.

The effect of perilla seed, oil and rumen‐protected oil supplementation to the diet on the composition of plasma fatty acids in the blood is illustrated in Table 8. The C8:0, C11:0, C12:0, C14:0, C15:0, C15:1, C16:1, C17:0 and C17:1 fatty acids were not affected by the treatments (*p* > 0.05). However, C16:0, C18:0 and *cis*‐9 C18:1 fatty acids decreased in perilla groups (*p* < 0.01). *Trans*‐11 C18:1, *cis*‐9, *trans*‐11 C18:1, C18:2 and C18:3n‐3 fatty acids increased in perilla groups (*p* < 0.01). C18:0 fatty acid was the lowest in the perilla seed and perilla rumen‐protected oil groups, whereas the highest was in the control group (*p* < 0.01).

**TABLE 8:** The effects of perilla seed, oil and rumen‐protected oil added to the diet on the composition of plasma fatty acids in the blood in lactating goats.

Fatty acids (g/100 g FAME)	CON	PS	PO	PPO	SEM	*p*
C8:0	3.60	4.21	4.18	3.90	0.21	0.124
C10:0	0.51	0.68	0.56	0.59	0.14	0.254
C12:0	0.48	0.71	0.65	0.66	0.08	0.604
C14:0	0.38	0.75	0.71	0.59	0.14	0.362
C15:0	0.44	0.51	0.48	0.49	0.18	0.441
C15:1	0.74	0.60	0.66	0.64	0.11	0.601
C16:0	19.47^a^	16.95^b^	17.41^b^	17.62^b^	0.65	0.001
C16:1	1.71	1.29	1.31	1.28	0.24	0.078
C17:0	0.69	0.61	0.64	0.62	0.14	0.395
C17:1	0.39	0.39	0.37	0.41	0.21	0.221
C18:0	23.68^a^	21.45^c^	22.14^b^	21.06^c^	0.14	0.000
*9* C18:1	21.84^a^	18.65^b^	18.78^b^	18.45^b^	0.71	0.003
*t*‐11 C18:1	0.01^b^	1.85^a^	1.71^a^	1.92^a^	0.74	0.000
*c*‐9, *t*‐11 C18:1	0.00^b^	0.01^a^	0.01^a^	0.01^a^	0.00	0.000
C18:2	22.45^b^	23.96^a^	24.05^a^	23.78^a^	1.09	0.000
C18:3n‐3	0.41^b^	4.65^a^	4.41^a^	4.79^a^	0.05	0.000

Abbreviations: CON, control, diet without oil; FAME, fatty acid methyl ester; PO, diet with perilla oil; PPO, diet with perilla rumen‐protected oil; PS, diet with perilla seed; SEM, standard error mean.

Values in the same row with superscript letters are significantly different at *p* < 0.05.

In the current study, the supplementation of perilla oil in different forms in the diet did not affect blood serum glucose, cholesterol or NEFA levels. Previously reported that serum triglyceride, LDL and HDL concentrations did not change, and serum glucose concentration increased with the supplementation of omega‐3‐rich sources in the diet (Kholif et al. 2020). Morsy, Kholif, and Kholif (2015) reported that the supplementation of sunflower seeds and oil to goat diets did not change blood chemistry but increased serum glucose concentration, which was associated with increased milk yield, and this was explained on the basis of the increase in total essential oil concentration in the rumen. Increasing NEFA concentrations indicate that fat is reducing because of increased energy demand, thereby suggesting that goats were in a negative energy balance due to increased nutrient demands for lactation (Ye et al. 2009). In the present study, plasma NEFA concentration did not change with changes in diet. Similarly, with the addition of 20 g/kg of flaxseed oil (Bu, Wang, and Dhiman 2007) and 40 g/kg of soybean oil (Ye et al. 2009) in the diet, LDL increased, whereas NEFA concentrations showed similar results.

In the current study, C16:0 and C18:0 fatty acids in blood plasma decreased in perilla groups, whereas rumenic and vaccenic acids and C18:3n‐3 fatty acids increased in perilla groups. In a study, sources of PUFA (soybean oil, flaxseed oil and fish oil, 20 g/kg DM) were used in the diet. It was reported that vaccenic acid and rumenic acids increased strongly in plasma concentrations in the diet using fish oil and that soybean oil and flaxseed oil supplementation changed the PUFA profile in blood plasma (Almeida et al. 2019). In a study conducted by adding protected and unprotected sunflower and flaxseed oil to the diet of beef cattle, it was reported that protected forms of sunflower and flaxseed oil increase blood plasma, linoleic acid, PUFA and PUFA/SFA levels compared to unprotected forms (Scislowski, Bauchart, and Gruffat 2005). In a study, it was determined plasma milk C18:3n‐3 ratios in cows fed ground flaxseed, formaldehyde‐treated linseed oil, extruded whole flaxseed and DHA‐fed flaxseed‐fed cows versus C18:3n‐3 in cows fed extruded flaxseed. Cows fed formaldehyde‐treated flaxseed oil showed higher plasma and milk C18‐3n‐3 fatty acids compared to other treatments (Sterk et al. 2011). However, Khiaosa‐ard, Kreuzer, and Leiber (2015) stated that the amount of linoleic and linolenic acids that pass through the rumen without undergoing biohydrogenation is very low, comparable to about 5% of the intake of these fatty acids in diet and milk. However, the higher concentration of UFA provided for blood plasma and milk can be attributed to the greater fat content in the diet, resulting in increased intestinal absorption of UFA and their transition from diet to milk. Ferreira et al. (2014) showed that the use of PUFA in the diet might decrease C18 fatty acid in plasma as it reduced ruminal biohydrogenation, increased duodenal flow and reduced SFA available for absorption.

## 4 Conclusion

Beneficial fatty acids can change largely through nutrition by increasing n‐3, CLA and PUFA concentrations in milk fat and decreasing SFA concentration without affecting performance, ruminal fermentation or blood parameters and appear to be improved by supplementation of perilla (*P. frutescens*) seed, oil or protected form (formaldehyde) to goat diets. In addition, these beneficial effects were found to be more pronounced with the use of protected (formaldehyde treatments) perilla oil in the diet. In addition, the supplementation of perilla as a seed, compared to its use as oil, has been effective in increasing both milk fat quality, so its use as a seed is thought to be more advantageous in terms of cost and storage.

## Author Contributions

**S.B.B**.: Conceptualization. **S.B.B. and İ.Ü**.: Material collation. **S.B.B. and B.O**.: Methodology. **S.B.B**.: Statistical analysis. **S.B.B., İ.Ü. and E.K**.: Resources. **S.B.B. and E.K**.: Writing–original draft preparation. **S.B.B., İ.Ü., E.K., B.O. and Y.K**.: Writing–review and editing. **S.B.B**.: Project administration. **S.B.B**.: Funding acquisition. All authors have read and agreed to the published version of the manuscript.

## Ethics Statement

The protocol for this study (19/087 no: 4) was approved by the Animal Experiments Local Ethics Committee of Erciyes University, Turkey.

## Conflicts of Interest

The authors declare no conflicts of interest.

## Data Availability

The data analysed in this investigation are available upon request to the corresponding author.
